# Strategies used to detect and mitigate system-related errors over time: A qualitative study in an Australian health district

**DOI:** 10.1186/s12913-024-11309-0

**Published:** 2024-07-24

**Authors:** Madaline Kinlay, Wu Yi Zheng, Rosemary Burke, Ilona Juraskova, Lai Mun Ho, Hannah Turton, Jason Trinh, Melissa T. Baysari

**Affiliations:** 1 Biomedical Informatics and Digital Health, School of Medical Sciences, Faculty of Medicine and Health, The University of Sydney, Sydney, NSW 2006 Australia; 2 Black Dog Institute, Sydney, Australia; 3 Pharmacy Services, Sydney Local Health District, Sydney, Australia; 4 School of Psychology, Faculty of Science, The University of Sydney, Sydney, Australia

**Keywords:** Electronic medical record, System-related errors, Patient safety, Error detection, Error prevention, Hospital

## Abstract

**Background:**

Electronic medical record (EMR) systems provide timely access to clinical information and have been shown to improve medication safety. However, EMRs can also create opportunities for error, including system-related errors or errors that were unlikely or not possible with the use of paper medication charts. This study aimed to determine the detection and mitigation strategies adopted by a health district in Australia to target system-related errors and to explore stakeholder views on strategies needed to curb future system-related errors from emerging.

**Methods:**

A qualitative descriptive study design was used comprising semi-structured interviews. Data were collected from three hospitals within a health district in Sydney, Australia, between September 2020 and May 2021. Interviews were conducted with EMR users and other key stakeholders (e.g. clinical informatics team members). Participants were asked to reflect on how system-related errors changed over time, and to describe approaches taken by their organisation to detect and mitigate these errors. Thematic analysis was conducted iteratively using a general inductive approach, where codes were assigned as themes emerged from the data.

**Results:**

Interviews were conducted with 25 stakeholders. Participants reported that most system-related errors were detected by front-line clinicians. Following error detection, clinicians either reported system-related errors directly to the clinical informatics team or submitted reports to the incident information management system. System-related errors were also reported to be detected via reports run within the EMR, or during organisational processes such as incident investigations or system enhancement projects. EMR redesign was the main approach described by participants for mitigating system-related errors, however other strategies, like regular user education and minimising the use of hybrid systems, were also reported.

**Conclusions:**

Initial detection of system-related errors relies heavily on front-line clinicians, however other organisational strategies that are proactive and layered can improve the systemic detection, investigation, and management of errors. Together with EMR design changes, complementary error mitigation strategies, including targeted staff education, can support safe EMR use and development.

**Supplementary Information:**

The online version contains supplementary material available at 10.1186/s12913-024-11309-0.

## Background

An electronic medical record (EMR) provides access to longitudinal patient data and clinical information in a timely and convenient manner, [1] while allowing clinicians to prescribe, review and administer medications on a single digital platform, often with the assistance of clinical decision support. Although the use of EMR systems results in fewer medication errors, [2] they can also create new system-related errors; errors that were highly unlikely or not possible with the use of paper medication charts (e.g. a doctor selecting the wrong dose from a drop-down menu). Previous research has identified the types and factors contributing to system-related errors, [3–5] as well as their prevalence [6], but the detection of these errors can be challenging in both a clinical and research context. Research investigating the types and rates of system-related errors at two hospitals revealed that of the 493 system-related errors that were discovered, only 13% were detected by hospital staff prior to the study [4]. Further, the rate of system-related errors varies between studies, ranging from 1.2 to 34.8% of all errors [7] with this rate dependent on the detection method employed [6]. 

To our knowledge, there has been no research that has specifically examined how system-related errors are detected by the organisations impacted by them. While the first step in reducing system-related errors is error detection, another important component of error management is learning from previous errors and improving on processes and systems [8, 9]. Our previous work has described EMR system enhancements made to target system-related errors, [10] however research on how system-related errors are rectified or managed once error detection has occurred is in its infancy. Therefore, the current study asked the following research questions: (1) what are the detection and mitigation strategies adopted by a health district to target system-related errors? and (2) what are stakeholder views on strategies needed to curb future system-related errors from emerging?

## Methods

### Context

This study formed part of a larger qualitative research project examining stakeholder understanding and experiences of system-related errors [11]. The research was conducted at three hospitals in Sydney, Australia, that used the same commercial EMR system (Cerner Millennium^®^). The system had been in place for different durations at each site (14 years, 4 years and 2 years) and roll-out strategies varied in length and approach.

### Recruitment and data collection

Participants included any hospital employee who dealt with the EMR directly or indirectly, including end-users (i.e., doctors, nurses, pharmacists), clinical informatics team members (e.g. system trainers), members of relevant committees (e.g. medicine safety committee) and department directors. A clinical informatics pharmacist at each site identified individuals who they believed were knowledgeable about the EMR or had relevant roles, and the research team invited these potential participants to take part via email. This technique was combined with snowball sampling, where participants were asked to propose additional staff members for inclusion. In total, 45 email invitations were distributed.

Semi-structured interviews were conducted either by video conference or in-person at the hospital. Interviews were in two parts. In Part 1, reported elsewhere, [11] participants were asked to describe common system-related errors and factors contributing to them. In Part 2, reported here, participants were asked to reflect on how system-related errors changed over time, and to describe detection and mitigation strategies their organisation had adopted. Separate interview guides were created for end-users and for individuals who supported EMR use (see the Additional file 1 and 2). Interview guides were developed by a multi-disciplinary team, including clinicians, and those with extensive knowledge of the EMR. Participants had the option to contact the researcher with any additional questions or comments following the interview. The lead investigator (MK), a student completing interviews as part of her doctoral degree, obtained written consent from participants and conducted all interviews. The interviewer was not known to participants before interviews commenced. Interviews were audio-recorded, transcribed verbatim and de-identified. Data collection ceased upon reaching thematic saturation across the overall dataset [12]. 

### Data analysis

Interviews were thematically analysed using a general inductive approach, where codes were assigned as themes emerged from the data [13]. Three researchers (MK, MB and WYZ) independently coded data from individual interviews into themes and met at regular intervals to discuss categories and resolve discrepancies. Data from the two different interview groups (end-users and individuals who supported EMR use) were analysed together, but general participant identifiers (users/EMR team) were maintained to allow any differences in the two groups to be identified. After agreeing upon a coding framework, researchers coded the remaining interviews and undertook a final review to discuss ambiguities, inconsistencies and confirm major themes and subthemes. Themes were checked by multi-disciplinary members of the research team, including clinicians and EMR experts, who confirmed face validity.

This project was approved by the district’s Human Research Ethics Committee (HREC reference number: 2020/ETH00198). All participants provided informed written consent to participate, including to be audio-recorded.

## Results

### Participant demographics

Interviews were conducted with 25 stakeholders, comprising 15 clinicians (end users of the EMR) and 10 staff from the EMR implementation and support team. Participant demographics appear in Table 1 (see [1] for more detailed demographics). Interviews occurred between September 2020 and May 2021 and took an average of 35 min, ranging from 9 to 55 min. No differences emerged in the results from end-users and individuals who supported EMR use, and therefore themes for these groups are presented together. Note that CI preceding a participant code (e.g. CIDR vs. DR) indicates the quotation relates to a clinical informatics (EMR) expert, not end-user.

**Table 1:** Participant demographics

Specialty	Total number (Number in EMR team)
Medical	5 (2)
Nursing	13 (5)
Pharmacy	7 (3)
*Years in current role*	
< 5 years	13 (5)
5–10 years	10 (5)
> 10 years	2 (0)

### Themes

An overview of the themes and subthemes, along with corresponding codes and quotations from interviews, is presented in Table 2.

**Table 2:** Codes used to develop themes with examples of quotations

Theme	Code	Quotation
**Detection of system-related errors**		
Clinicians detect error	Nurse identifies error (i.e. during routine checks)	*‘Nursing staff will also check orders and before administering medications*,* and they may recognize one of these system errors.’* (CIDR2)
	Pharmacist identifies error (i.e. during medication review)	*So as pharmacists*,* the main way we pick up errors is when we review medications; medication orders on the MAR.’* (PH2)
Organisational processes to detect errors	Clinicians report error to clinical informatics team	*‘I come across system errors all the time and*,* for the most part*,* I’ll report them to our health informatics team’* (NU8)
	Clinicians lodge report in the incident information management system	*‘These types of errors would be identified by clinicians*,* self-reporting these medication incidents. So they would report through the normal IIMS process […] that there’s been a medication error.’* (CINU2)
	Clinical informatics team run EMR report	*I will run reports on EMR to see whether this is a consistent pattern that is happening across the facility and if is consistent across the facility*,* then this is definitely an issue that we need to address.’* (CIPH2)
	Adverse patient event investigation	*‘An error that’s caused something adverse to happen to a patient; it might be raised in that manner.’* (CINU3)
	System improvement project	*‘I’m also aware from my IT job that it often comes up in testing of new functionality. So we might identify that we’ve got a system related error in the way downtime medications are displayed.’* (CIDR2)
**Management and mitigation of system-related errors**		
EMR design changes	Clinical informatics team modify system design or set up	*‘So if there’s a system related error*,* we will escalate it for changes.’* (CIPH3)
Organisational strategies to manage and mitigate errors	Individual education or feedback	*‘There’s individual education […] We will investigate first*,* then we’ll go and see the person*,* and go through the steps that have caused it.’* (CINU4)
	Group or hospital-wide education	*‘It’s very hard to change those things. It’s about education to make sure that people have an understanding about the therapeutics involved.’* (PH3)
**System-related errors over time**		
How system-related errors change	Only some errors are eliminated	*“There’s always going to be the ones that we can’t resolve*,* in that we can’t change […] the system.”* (CINU1) *“Well*,* ones we’ve identified*,* I think*,* have improved.”* (CINU3)
	The types of errors change	*“Number of errors hasn’t changed*,* but the distribution of types of errors might be a bit different.”* (PH3)
	Fewer errors over time	*“I would say error rates do decrease as people become more familiar with the system.”* (CIDR1)
Why system-related errors change	New EMR functionality/safety features added	*“As we continue to change it and change the workflows*,* we will get different errors.”* (CIPH3)
	Users become more familiar with the EMR system	*“I think it’s like*,* with experience*,* you kind of learn how to use [the EMR] and how to avoid kind of errors. But obviously you would have had to have made some errors […] and then learn from them.”* (DR2)
	Users start to take shortcuts/workarounds	*“When you’re not familiar with the system*,* you try to chart everything from scratch but when you’re familiar with the system*,* you kind of take certain shortcuts.”* (CIPH2)
	More widespread EMR use	*“A lot of the students that are coming through and become graduates*,* all they’ve ever known is [EMR] and so they’ve become used to it.”* (CINU5)
	New staff members	*“When new people start*,* sometimes we get a little bit of a resurgence in [system-related errors].”* (CINU4)
	Staff are no longer familiar with paper systems	*“Some of the new young generation they find difficult to use as a paper form when a downtime happen.”* (NU5)
	Targeted education provided to users	*“We’ve trained our nurses*,* and we’ve trained our pharmacists to be on a particular lookout for these kinds of prescribing errors.”* (PH1)

IIMS = incident information management system, MAR = medication administration record, EMR = electronic medical record, CI = clinical informatics, PH = pharmacist, NU = nurse, DR = doctor

### Detection of system-related errors

**Fig. 1:**
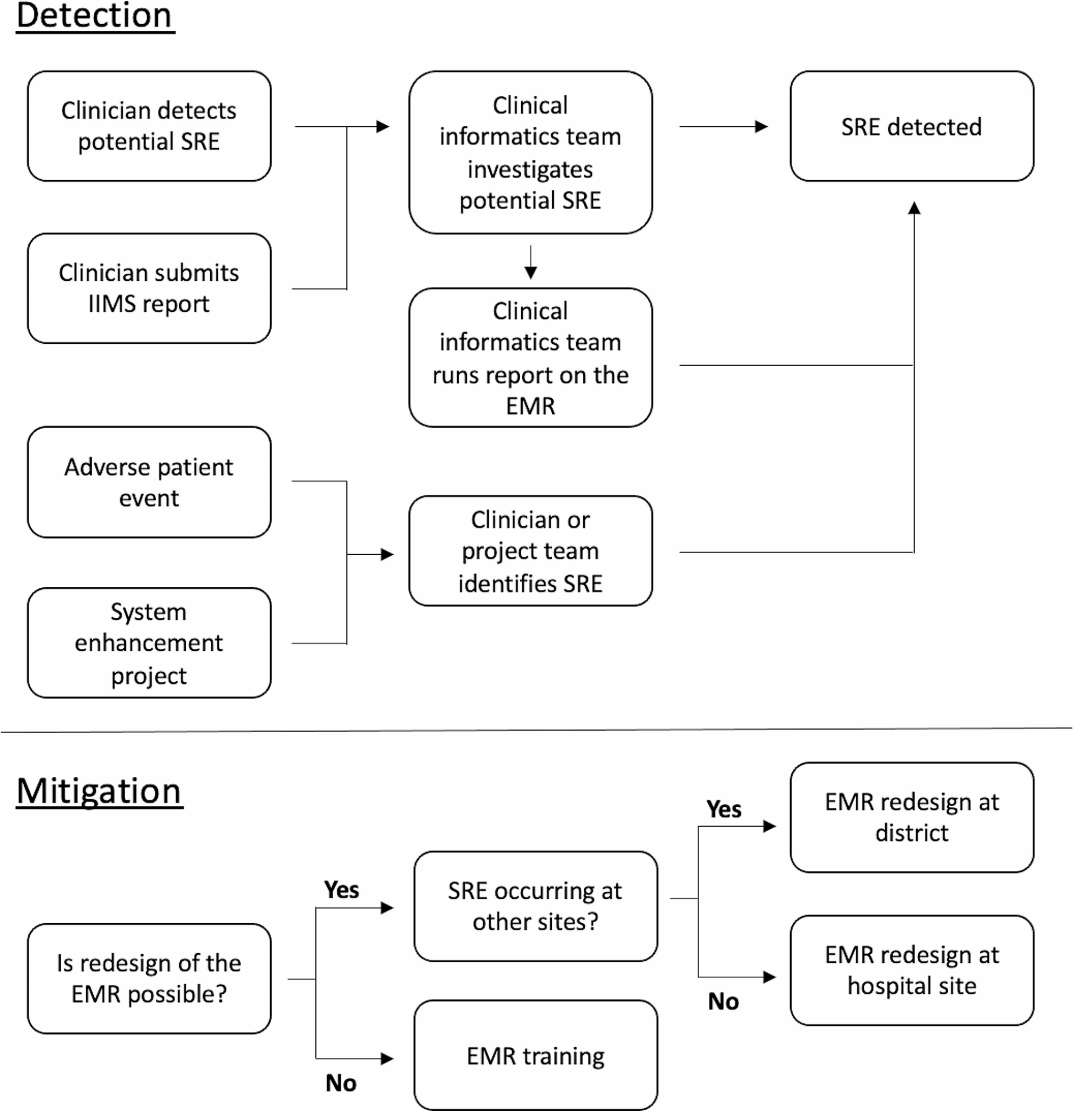
Flowchart depicting the process by which system-related errors are detected and mitigated by hospital staff, based on the themes extracted from interviews with key stakeholders. SRE = System-related error, IIMS = Incident information management system, EMR = Electronic medication record

Participants described several methods by which system-related errors were detected by the hospital sites (see Fig. 1).

#### Detection of system-related errors by clinicians

Detection by front-line clinicians was the primary method of system-related error detection reported by participants. Specifically, participants explained that pharmacists identified system-related errors during medication review or reconciliation, and nurses detected system-related errors when completing routine checks prior to administering medications. *‘All orders get verified by a pharmacist*,* so that pharmacist might intervene if they recognise that an error has occurred by reviewing the order. And nursing staff will also check orders and before administering medications*,* and they may recognise one of these system errors.’* (CIDR2).

However, some participants noted that detecting system-related errors was often difficult for nurses as it required them to discern the intended prescription from the recorded prescription.

#### Organisational processes in place to detect system-related errors

One of the most frequent organisational strategies highlighted by participants to complement clinicians’ detection of system-related errors was clinicians reporting potential system-related errors to the clinical informatics team, who then ascertained whether the error was in fact system-related. Clinical informatics team members noted that system-related errors were difficult to detect without clinician input, and investigations into system-related errors were often dependent on clinicians bringing potential cases to their attention. *‘Frankly speaking*,* you don’t have anything that can alert you […] It requires a lot of clinicians reporting these issues back to me*,* for me to be able to know these things are happening on the ward.’* (CIPH2).

Participants also explained that system-related errors could be detected via the Incident Information Management System (IIMS); the organisation’s voluntary reporting system for clinical, work health and safety, and security events. *‘So*,* at a high level they can be reported through our incident monitoring system.’* (PH3).

However, interviewees also noted that this detection strategy relied upon clinicians identifying and proactively self-reporting system-related errors. *‘In terms of how we found out about them*,* incident reporting is something I think we are hoping to be more and more proactive about.’* (CIDR2).

Another method reportedly used by clinical informatics staff to detect system-related errors was the generation of specific reports within the EMR, such as a monthly report of pharmacy interventions to identify reports that cited the involvement of an EMR system issue. These reports displayed trends in error types and were viewed as useful for determining whether specific system-related errors occurred regularly and what factors could be contributing to error occurrence. *‘I will run reports on the EMR to see whether there is a consistent pattern that is happening across the facility. […] Identifying patterns*,* identifying whether it’s a prescribing issue or whether it’s a nursing workflow issues*,* or whether it is actually an EMR issue.’* (CIPH2).

Some participants reported that errors were detected by a clinician or project team during inquiries into adverse patient events or during EMR system enhancements when intensive testing sometimes uncovered system-related errors. For instance, when creating a new cancer module in the EMR, project team members discovered that chemotherapy prescriptions did not display all the necessary order components to the user.

### Management and mitigation of system-related errors over time

Participants described various approaches to manage and reduce system-related errors, including EMR design changes and organisational strategies (see Fig. 1).

#### EMR design changes to mitigate system-related errors

Participants explained that after clinicians escalated concerns to the clinical informatics team and a system-related error was confirmed, the EMR system design was modified, if this was deemed to be essential and possible. Modification of the EMR system design could occur when the clinical informatics team recognised a patient safety or workflow benefit from the change and the system was able to be altered (i.e. no system configuration limitations). *‘Where we have found people making mistakes*,* we’ve been able to implement some actions to circumvent them.’* (PH3).

Looking forward, participants stated that over time they would expect fewer system-related errors, attributing this reduction to the fact that errors had been identified and rectified.

*‘Because*,* one*,* we are better aware of how to design the system to reduce the likelihood of some of these errors.’* (CIDR2).

Participants provided specific examples of system redesign to target system-related errors (see Table 3). A frequently reported category of system redesign was the addition of alerts for specific processes and medications, such as high-risk medications. Improved visibility and clarity of information in the EMR was another strategy reported by participants to mitigate system-related errors. Participants also described a more intuitive and consistent system. References were made to incorporating human factors design principles into the EMR and ensuring the system aligns with workflow. For example, one doctor suggested that the system become more user-friendly when adjusting doses and times, while a pharmacist proposed that the system provide more clarity of the job role required so that clinicians know which tasks to attend to on the system (i.e., checking off a box is only for nurses).

Although EMR design changes were said to decrease system-related errors, participants highlighted that it was possible for these system functionality changes to result in new types of errors over time.

*‘As we continue to change it and change the workflows*,* we will get different errors’* (CIPH3).

Participants also noted that some current system-related errors would remain, citing constraints in the system build, preventing design changes that could resolve errors and therefore requiring other strategies to manage these system-related errors. *‘There’s always going to be the [errors] that we can’t resolve*,* in that we can’t change the way the system is built’* (CINU1).

**Table 3:** Specific examples of design changes made to the EMR described by participants to reduce system-related errors

EMR design change	Rationale	Quote
**Former EMR changes made to reduce system-related errors**		
Addition of an alert to notify doctors that the default medication time had been prescribed	• To ensure the time of the medication was reviewed prior to finalising a prescription • To reduce timing errors associated with the use of antibiotics	*‘The system now will pop up and say to you that oh*,* you know*,* the antibiotic that you’ve prescribed*,* the start time is more than an hour away. Is this intentional? Or do you want to change the order?’* (PH1)
Addition of a duplication alert for high-risk medications, such as anticoagulants	• To minimise the risk of a patient receiving two medications from the same therapeutic class; an error that was said to result from patients’ current medications not appearing on the EMR screen while prescribing	*‘So now we have what we call a dual anticoagulant pop-up alert. What I mean is*,* for example*,* if the patient has already been prescribed a blood thinning medication and the doctor attempts to prescribe another one*,* they will get a pop-up alert notifying them that*,* you know*,* “you have got a blood thinning agent already prescribed.”’* (CIPH2)
Forcing functions for high-risk medications, such as hydromorphone	• To ensure clinicians reviewed medication parameters selected as part of an order sentence, such as dosage, prior to prescribing or administering medications	*‘They get a pop-up alert to confirm that they are wanting to prescribe hydromorphone*,* and the dose that they are wanting to prescribe*,* to try and prevent overdoses of that medication.’* (CINU2)
Introduction of Tallman lettering	• To reduce the risk of selection errors by making medications that sound and look similar more distinguishable from one another	*‘There’s a lot of work that’s been done in relation to*,* you know*,* Tallman lettering and all that kind of stuff*,* to make sure that the medicines with a similar name etc are better identified in the EMR.’* (CINU5)
Change made to the display of the medication warfarin	• To improve visibility and decrease fragmentation of the warfarin order	*‘There was a prescribing and administration issue with warfarin. […] So we fixed that up so that you could actually see the order details in the right chronological order rather than it being a bit fragmented’* (CINU5)
**Recommended changes to the EMR to reduce system-related errors in the future**		
Redesign of existing alerts, such as the wording, layout, and complexity of alerts	• To improve their effectiveness in reducing system-related errors	*‘If you’ve got multiple alerts at the moment*,* you get each one individually*,* and you have to review them. Newer designs will lay them all out and you can make decisions about each of them within one screen*,* less clicks*,* less movement and a cleaner interface.’* (CIDR2)

EMR = electronic medical record, CI = clinical informatics, PH = pharmacist, NU = nurse, DR = doctor

#### Organisational strategies to mitigate system-related errors

The most frequently reported organisational strategy employed to minimise system-related errors was education, either to an individual user, a group of clinicians, or hospital-wide. Providing individual feedback or training was said to occur in response to a specific incident, usually in cases where unfamiliarity with the EMR was believed to have contributed to the error. When system-related errors were more widespread, occurring across a particular cohort, ward or hospital, participants explained that education was delivered more broadly.

*‘Once [nurses] have flagged the problem to the helpdesk*,* the supervisor or whoever’s in charge*,* […] they will try to find the problem and then give us advice on what to do next.’* (NU7).

Participants referred to examples where system functionality or configuration was unable to be changed after identification of a system-related error, and so staff education and training focused on safely bypassing system limitations or constraints so that work could continue.

Although education was viewed to be an effective strategy for reducing system-related errors, some participants reported the challenge of system-related errors persisting due to staff turnover and the employment of new clinicians.

*‘Because its constantly new staff coming in*,* they then don’t know the messages that have been sent out last year… They tend to make the same mistake again at some point or another.’* (PH3).

However, participants explained that with more widespread EMR use in the future, users would become more familiar and confident with the system, and fewer system-related errors would result. Despite this, new errors were reported to also arise when users take more shortcuts or workarounds as they become more familiar with the system. For example, a clinical informatics pharmacist described clinicians exporting information from previous admissions into the patient’s current medication chart without consulting the patient.

*‘You’re seeing different types of errors where prescribers are very comfortable now with using information from previous admissions but forgetting that they also need to talk to patient and get updated information … When you’re familiar with the system*,* you kind of take certain shortcuts.’* (CIPH2).

Some clinical informatics team members noted that raising issues with the chief executive or chief information officer was another organisational strategy used to mitigate system-related errors, particularly when system-related errors were likely to be occurring at other hospital sites and system changes at a broader level were necessary.

Finally, minimising the use of hybrid systems (i.e., paper and electronic systems, dual electronic systems), was mentioned by some participants as another strategy to reduce system-related errors. However, participants also noted that as users become less familiar with paper-based medication charts, new errors may arise when clinicians are required to use paper charts during EMR downtime. *‘Some of the new*,* younger generation*,* they find it difficult to use as a paper form*,* when a downtime happens.’* (NU5).

## Discussion

Interviews uncovered detection and mitigation strategies implemented by a health district to target system-related errors, including existing and potential methods required to prevent future errors from occurring. Participants explained that initial detection of system-related errors was highly dependent on clinicians identifying errors. Once error detection occurred, participants highlighted that clinicians either directly reported these errors to the clinical informatics team or submitted an IIMS report for escalation. EMR redesign was described as the main approach for error reduction, however other organisational strategies, like regular user education and minimising the use of hybrid systems were also reported.

It is noteworthy that many of the reported approaches for system-related error detection put the onus on clinicians to identify and subsequently report errors. Although verbal and incident reporting by clinicians are conventional methods of error detection, irrespective of EMR involvement, [14] system-related errors are challenging for clinicians to recognise and may go unnoticed unless they lead to an error (i.e. medication error) or adverse patient event [15]. Clinicians’ reliance on the EMR system for care delivery is growing due to an increase in automation and system guidance, [16, 17] influencing their ability to recognise a system-related error. Additionally, the complexity of the EMR system, [18] unfamiliarity with the EMR, and distraction caused by competing priorities [19] can all hinder detection of system-related errors.

In addition to difficulties in error detection, challenges associated with reporting of system-related errors are also likely. Clinicians may not report system-related errors if they fear individual blame or punishment, [20] or are unsupported in their efforts to improve patient safety [21, 22]. Factors driving under-reporting of incidents are likely to also be at play in reporting of system-related errors to clinical informatics teams, including a perception of low value of reporting if reports are not used to identify error patterns and prevent future incidents [23]. 

Implementing a systematic feedback process, where clinicians are informed of changes to EMR systems or processes that result from reporting, would increase the perceived value, confidence and motivation to report system-related errors. The challenges associated with clinician detection and reporting of system-related errors highlight the importance of utilising complementary strategies to detect these errors. We found that system enhancement projects, as well as EMR reports, were other proactive methods of detection, though reported less often. Combining reactive front-line detection with proactive clinical surveillance and monitoring is likely to ensure system-related errors are promptly identified and investigated [15]. 

EMR design changes were the most common approach suggested by participants to reduce system related errors, with many believing EMR redesign would result in fewer system-related errors. However, an unintended consequence of modifying system configuration was the generation of different system-related errors, and several participants stated that certain errors would persist as constraints in the EMR system build limited design alterations. While incremental design changes are necessary for maintenance and development of the EMR system, [24] the possibility of design changes resulting in the emergence of different system-related errors reinforces the importance of testing environments that simulate real-life EMR situations prior to the go-live of any modifications [25]. 

Education, either one-on-one, to a particular cohort, or hospital wide, was another mitigation strategy we identified to reduce system-related errors. Despite the reported benefits of education, participants noted that staff turnover and the employment of new staff could contribute to an increase in errors. By regularly updating training material and providing periodic, targeted education (e.g. as part of onboarding new staff), this would ensure new staff are aware of the most up-to-date material and minimise the risk of medication errors [26]. Participants indicated that as more staff become proficient in using EMRs, there are likely be fewer system-related errors, but potentially larger numbers of workarounds, with previous research supporting this latter suggestion [27, 28]. Although workarounds can compromise patient safety and quality of care, [29] comprehensive training about EMR risks and ongoing support for EMR users, can reduce clinicians’ use of workarounds [3]. 

### Strengths and limitations

Qualitative research methods allowed the authors to conduct, for the first time, an in-depth investigation of detection and mitigation strategies, however this research did not measure how often system-related errors were detected or the effectiveness of improvement methods. Additionally, interviews were conducted with clinicians and key stakeholders in one Local Health District, across only three hospitals, and therefore results may not be generalisable to other settings and the detection and mitigation approaches identified may not be exhaustive.

## Conclusions

To our knowledge, this is the first study to examine how system-related errors are detected by organisations and adds to the growing body of evidence exploring error mitigation. Front-line clinicians play a critical role in system-related error detection, however other organisational approaches, such as system enhancement projects, improve systemic error detection, investigation, and management. Organisations must take a proactive approach to error identification and ensure detection processes are layered. Although EMR design changes were highlighted as important for error reduction, changes were not always possible. Complementary strategies, such as targeted staff education, can support safe use of the EMR and its ongoing development.

### Electronic supplementary material

Below is the link to the electronic supplementary material.

Supplementary Material 1

## Data Availability

The qualitative data collected from participants for this study are not available.

## Abbreviations

EMR: Electronic medical record
MAR: Medication Administration Record
IIMS: Incident Information Management System
CI: Clinical Informatics
PH: Pharmacist
NU: Nurse
DR: Doctor
