# Supplementary material for: Strategies used to detect and mitigate system-related errors over time: A qualitative study in an Australian health district
Source: BMC Health Serv Res. 2024 Jul 24;24:839. doi: 10.1186/s12913-024-11309-0 (PMC11270877; doi:10.1186/s12913-024-11309-0)
Supplement: Supplementary file 1 — Supplementary Material 1 [file 12913_2024_11309_MOESM1_ESM.docx]

**SUPPLEMENTARY MATERIAL**

**Additional file 1: Semi-structured interview questions for hospital staff members/clinicians directly using the EMR**

**Basic demographics**

1. What type of staff member/clinician are you (i.e. nurse, doctor, pharmacist) and roughly how many years have you been in this role?
2. What ward/specialty do you predominantly work in?

**About EMR**

1. How long have you been using the *EMR* system?
2. Did you have experience with paper-based medication systems prior to using the *EMR*?
3. How often do you use the *EMR*? For example, to prescribe.
4. What training did you receive on the *EMR*?
5. How proficient do you feel you are in using the *EMR*?

**System-related errors**

*As mentioned, I’m interested in system-related errors of the EMR.*

1. How would you define a system-related error?

*In my study, I am defining a system-related error as any error that was unlikely or unable to occur in paper-based medication management systems. In other words, these errors would be unlikely or not possible without the EMR.*

Using this definition:

1. How are system-related errors identified or detected in the hospital?
2. Once a system-related error is detected, how are these errors managed or rectified?

**Timing of system-related errors**

*In thinking about the types of system-related errors that happen now:*

1. Do you think they were different to the types of errors that occurred when the system was first implemented (e.g. during the first 6-months of use?)
2. Do you think these errors will be different in the future, once using the system becomes routine/as the system continues to be used?

**Future of the EMR**

1. In your opinion, what interventions or changes have improved the system or reduced system-related errors?
2. What would you like to see change about the system?
   1. Why?
3. Do you have anything further to add that has not already been discussed?

**Additional file 2: Semi-structured interview questions for all other stakeholders**

**Basic demographics**

1. What is your current role and roughly how many years have you been in this role?
2. What interactions do you have with the *EMR*?

**About EMR**

1. How long have you been supporting the use of the *EMR* system?
2. What training did you receive on the *EMR*?

**Detection of system-related errors**

*As mentioned, I’m interested in the system-related errors of the EMR.*

1. How would you define a system-related error?

*In my study, I am defining a system-related error as any error that was unlikely or unable to occur in paper-based medication management systems. In other words, they would be unlikely or not possible without the EMR.*

Using this definition:

1. How are system-related errors identified or detected in the hospital?
2. Once a system-related error is detected, how are these errors managed or rectified?

**Timing of system-related errors**

*In thinking about the types of system-related errors that happen now:*

1. Do you think they were different to the types of errors that occurred when the system was first implemented (e.g. during the first 6-months of use?)
2. Do you think these errors will be different in the future, once using the system becomes routine/as the system continues to be used?

**Future of the EMR**

1. In your opinion, what interventions or changes have improved the system or reduced system-related errors?
2. What would you like to see change about the system?
   1. Why?
3. Do you have anything further to add that has not already been discussed?

**Additional file 3: COREQ (COnsolidated criteria for REporting Qualitative research) Checklist**

| **Topic** | **Item No.** | **Guide Questions/Description** | **Reported on Page No.** |
| --- | --- | --- | --- |
| **Domain 1: Research team and reflexivity** | | | |
| *Personal characteristics* | | | |
| Interviewer/facilitator | 1 | Which author/s conducted the interview or focus group? | 6 |
| Credentials | 2 | What were the researcher’s credentials? E.g. PhD, MD | 6 |
| Occupation | 3 | What was their occupation at the time of the study? | 6 |
| Gender | 4 | Was the researcher male or female? | NA |
| Experience and training | 5 | What experience or training did the researcher have? | 6 |
| *Relationship with participants* | | | |
| Relationship established | 6 | Was a relationship established prior to study commencement? | 6 |
| Participant knowledge of the interviewer | 7 | What did the participants know about the researcher? e.g. personal goals, reasons for doing the research | 6 |
| Interviewer characteristics | 8 | What characteristics were reported about the interviewer/facilitator? e.g. Bias, assumptions, reasons and interests in the research topic | 6 |
| **Domain 2: Study design** | | | |
| *Theoretical framework* | | | |
| Methodological orientation and Theory | 9 | What methodological orientation was stated to underpin the study? e.g.  grounded theory, discourse analysis, ethnography, phenomenology, content analysis | 6 |
| *Participant selection* | | | |
| Sampling | 10 | How were participants selected? e.g. purposive, convenience, consecutive, snowball | 5 |
| Method of approach | 11 | How were participants approached? e.g. face-to-face, telephone, mail, email | 5 |
| Sample size | 12 | How many participants were in the study? | 7 |
| Non-participation | 13 | How many people refused to participate or dropped out? Reasons? | 7 |
| *Setting* | | | |
| Setting of data collection | 14 | Where was the data collected? e.g. home, clinic, workplace | 6 |
| Presence of nonparticipants | 15 | Was anyone else present besides the participants and researchers? | 6 |
| Description of sample | 16 | What are the important characteristics of the sample? e.g. demographic data, date | Table 1 |
| *Data collection* | | | |
| Interview guide | 17 | Were questions, prompts, guides provided by the authors? Was it pilot tested? | 5, Appendix A and B |
| Repeat interviews | 18 | Were repeat interviews carried out? If yes, how many? | N/A |
| Audio/visual recording | 19 | Did the research use audio or visual recording to collect the data? | 6 |
| Field notes | 20 | Were field notes made during and/or after the interview or focus group? | N/A |
| Duration | 21 | What was the duration of the interviews or focus group? | 7 |
| Data saturation | 22 | Was data saturation discussed? | 6 |
| Transcripts returned | 23 | Were transcripts returned to participants for comment and/or | 5 |
| **Domain 3: analysis and findings** | | | |
| *Data analysis* | | | |
| Number of data coders | 24 | How many data coders coded the data? | 6 |
| Description of the coding tree | 25 | Did authors provide a description of the coding tree? | Figure 1 |
| Derivation of themes | 26 | Were themes identified in advance or derived from the data? | 6 |
| Software | 27 | What software, if applicable, was used to manage the data? | N/A |
| Participant checking | 28 | Did participants provide feedback on the findings? | 5 |
| *Reporting* | | | |
| Quotations presented | 29 | Were participant quotations presented to illustrate the themes/findings?  Was each quotation identified? e.g. participant number | 7-12 |
| Data and findings consistent | 30 | Was there consistency between the data presented and the findings? | 7-12 |
| Clarity of major themes | 31 | Were major themes clearly presented in the findings? | 7-12 |
| Clarity of minor themes | 32 | Is there a description of diverse cases or discussion of minor themes? | 7-12 |

Developed from: Tong A, Sainsbury P, Craig J. Consolidated criteria for reporting qualitative research (COREQ): a 32-item checklist for interviews and focus groups. *International Journal for Quality in Health Care*. 2007. Volume 19, Number 6: pp. 349 – 357
